# Immune Response and Transcriptome Analysis of the Head Kidney to Different Concentrations of *Aeromonas veronii* in Common Carp (*Cyprinus carpio*)

**DOI:** 10.3390/ijms252212070

**Published:** 2024-11-10

**Authors:** Jin Zhang, Ning Ding, Yingjie Qi, Na Jiang, Wei Xing, Tieliang Li, Zhihong Ma, Yiming Cao, Yan Zhang, Jiongtang Li

**Affiliations:** 1 Key Laboratory of Aquatic Genomics, Ministry of Agriculture and Rural Affairs and Beijing Key Laboratory of Fishery Biotechnology, Chinese Academy of Fishery Sciences, Beijing 100141, China; zhangjin@cafs.ac.cn (J.Z.); 18842022137@163.com (N.D.); caoyiming@cafs.ac.cn (Y.C.); 2 Chinese Academy of Agricultural Sciences, Beijing 100081, China; 3 National Demonstration Center for Experimental Fisheries Science Education, Shanghai Ocean University, Shanghai 201306, China; qyj521720@163.com; 4 Fisheries Science Institute, Beijing Academy of Agriculture and Forestry Sciences, Beijing 100068, China; lidiaj0@126.com (N.J.); xingwei2008cool@163.com (W.X.); litieliang8090@126.com (T.L.); mzh255@sohu.com (Z.M.)

**Keywords:** *Aeromonas veronii* infection, common carp, immune response, transcriptome, DEGs, WGCNA

## Abstract

The common carp (*Cyprinus carpio*), a major economic freshwater fish, is suffering from a variety of bacterial infectious diseases because of its high-density, factory and intensive farming patterns. *Aeromonas veronii* is the causative agent of high mortality in common carp, causing severe economic losses in aquaculture. However, the regulatory mechanisms involved in the response of common carp to this bacterial infection remain poorly understood. In this study, we compared mortality, blood serum LZM (Lysozyme) and IgM (Immunoglobulin M) levels and transcriptome patterns of head kidney tissues after infection with different concentrations of *Aeromonas veronii*. We observed that mortality increased progressively with an increasing pathogen concentration. The concentrations of blood serum LZM and IgM significantly increased after infection. A total of 13 and 925 differentially expressed genes (DEGs) were identified after infection with low (T4) and high (T9) concentrations of bacterial suspension, respectively. KEGG and GO analyses of the DEGs highlighted multiple immune-related signaling pathways. Weighted gene co-expression network analysis (WGCNA) revealed that 136 and 83 hub genes were related to blood serum LZM and IgM, respectively. Finally, the gene expression in the head kidney was validated via RT–qPCR to be consistent with the transcriptome. These results provide insights into the mechanisms of the immune response to infection with different concentrations of *Aeromonas veronii* and offer useful information for further studies on immune defense mechanisms in common carp.

## 1. Introduction

The common carp (*Cyprinus carpio*) is a prominent freshwater species that is widely distributed throughout the world, thriving in a vast array of environmental conditions due to its remarkable adaptability [1,2,3]. The common carp has a long domestication and cultivation history in China and not only plays an important role in the traditional Chinese culture but also is a delicacy of people’s tables, especially in northern China [4,5]. External environmental stresses pose a threat to the healthy farming of common carp by weakening their immune system and increasing their sensitivity to various pathogens [6,7]. In addition, high-density artificial farming and deterioration of water quality exacerbated the rapid spread of diseases, resulting in large−scale economic losses. It is an urgent issue that needs to be addressed to reduce the risk of disease and improve the overall amount of healthy common carp farming.

*Aeromonas veronii*, Gram−negative parthenogenetic anaerobic bacilli, is a type of *Aeromonas* that is a critical pathogen in the aquaculture industry and is especially widespread in freshwater [8,9]. It is an opportunistic pathogen in fish that causes bacterial infectious diseases, such as septicemia, hemorrhage, ulcers and ascites, and high mortality in diseased fish, resulting in considerable economic losses in aquaculture and posing a serious risk to fisheries worldwide [10,11,12]. Notably, this bacterium can produce zoonotic diseases through aquatic products, and its harm as an aquatic zoonotic agent has been demonstrated by an increasing number of cases [13,14,15]. Many studies have focused on the mechanisms of resistance to *Aeromonas veronii* infection in fish. The pathogenesis of this bacterium depends mainly on its virulence genes and virulence factors. The strain SJ4 has been reported to cause cellular enlargement, marked hemorrhage and inflammatory responses in diseased *Siniperca chuatsi*, and SJ4 not only carried virulence genes such as *act*, *fim*, *flgM*, *ompA*, *lip*, *hly*, *aer* and *eprCAL*, but produced tyrosinase, dnase, gelatinase and hemolysin [16]. Changes in the expression levels of host-associated immune genes are key mechanisms involved in the response to the infection. *Aeromonas veronii* could cause mass mortalities in *Odontobutis potamophila*, and the immune-related gene expression levels of *MHC II*, *Myd88*, *TLR* and *SOD* were significantly increased in the liver, gill, spleen and head kidney at different infection times [17]. A total of 114 immune−related DEGs were captured in the spleen transcriptome profiles of African catfish (*Clarias gariepinus*) challenged with this bacterium and were significantly enriched in 38 pathways related to immunity or disease, including the NF-kappa B, TNF, NLR, TLR and RLR pathways [18]. In addition, key genes involved in resistance to its infection in certain fish species have been identified, such as galectin−9, which was involved in the immune response against infection in koi carp (*Cyprinus carpio*) [19]; the *CD4-1* gene, which activated NF−κB signaling in response to infection [20]; and the mRNA expression levels of *CaCD3γ/δ*, which significantly changed in the spleen, head kidney, intestine and gill after infection in Qihe crucian carp (*Carassius auratus*) [21].

To explore the molecular mechanisms underlying the defense against *Aeromonas veronii* infection, we compared the transcriptome profile differences between head kidney tissues from control carp and infection groups with different concentrations of bacterial suspension and identified immune-related genes and signaling pathways activated or suppressed during this pathogen infection. Understanding the mechanisms of the immune response to *Aeromonas veronii* in common carp is crucial for controlling and preventing infections in aquaculture development strategies. Ultimately, this research facilitates the development of scientifically informed approaches to increase the disease resistance of freshwater aquaculture species.

## 2. Results

### 2.1. Comparison of Morbidity and Mortality at Six Infection Concentrations

**Figure 1:**
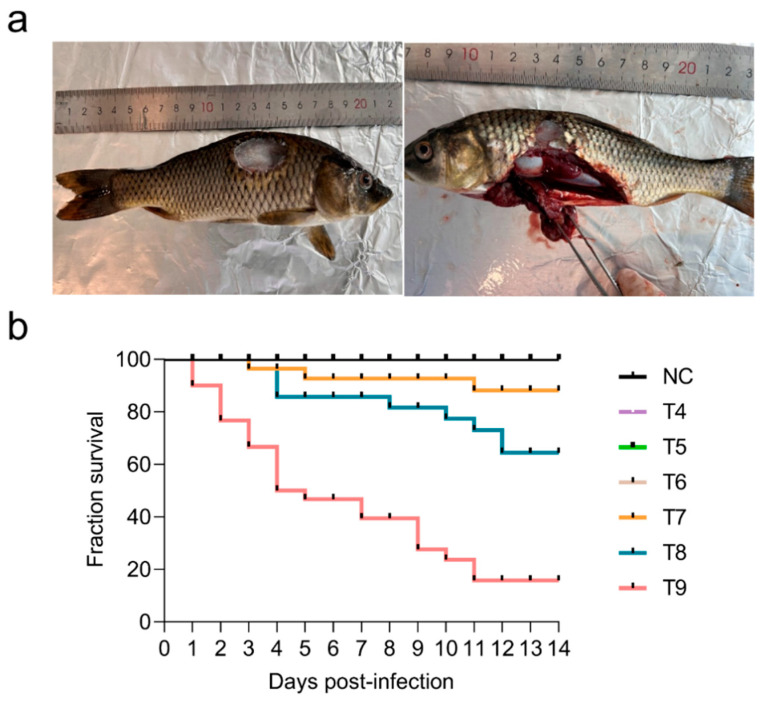
Symptoms of diseased fish and survival curves of common carp in the six groups challenged with different concentrations of *Aeromonas veronii*. (**a**) Symptoms of diseased fish: the left image shows the skin ulceration phenotype, and the right image shows the abdominal anatomy; (**b**) Survival curves of common carp in the six groups challenged with different concentrations of *Aeromonas veronii*. The four lines of NC, T4, T5 and T6 coincided.

Within 14 days of *Aeromonas veronii* infection, we counted morbid individuals, dead individuals and the time of death in the six groups with different infection concentrations. Except for the T4 group, the other five infection groups included morbid individuals with obvious symptoms, including skin ulceration, hemorrhages on the body surface, and intestinal dropsy (Figure 1a). In the T5 and T6 groups, only two morbid individuals were observed. Nevertheless, a total of 22 morbid individuals and three dead individuals were observed in the T7 group, and the morbidity and mortality were 73.33% and 10.00%, respectively. The highest morbidity of 96.67% was recorded in the T8 and T9 groups, and the mortality reached 80.00% in the T9 group. Overall, morbidity and mortality increased with increasing concentrations of *Aeromonas veronii* infection, especially in the high-concentration T9 group, compared with those in the low-concentration T4 group (Table 1 and Figure 1b). Notably, three acutely dead individuals in the T9 group died at 1 day post infection due to high concentrations of *Aeromonas veronii* (Supplementary Table S1).

### 2.2. Blood Serum LZM and IgM Responses to Different Infection Concentrations

**Figure 2:**
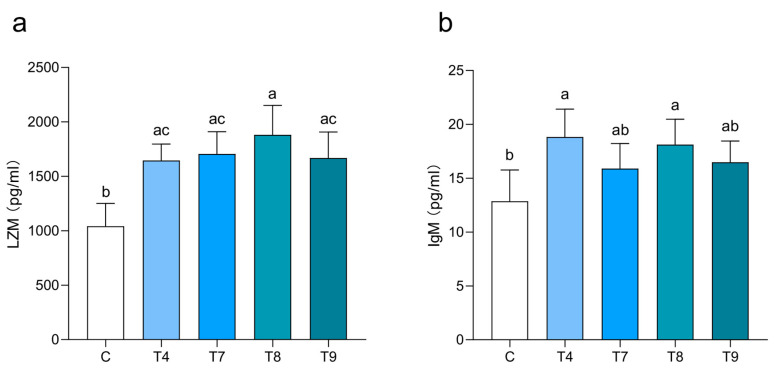
Comparison of the serum LZM and IgM concentrations in common carp challenged with different infection concentrations. (**a**) Concentration differences of LZM in different groups; (**b**) Concentration differences of IgM in different groups. The five groups of data with different letters indicate significant differences in the figure (*p* ≤ 0.05).

To assess the immune response to *Aeromonas veronii* infection, the concentrations of blood serum LZM and IgM in common carp were compared across four representative groups with different concentrations of infection. We selected the samples in the low-concentration T4 group (*n* = 10) and the high-concentration T7 (*n* = 6), T8 (*n* = 10) and T9 (*n* = 6) groups. In the noninfected group, the blood serum LZM concentration was greater than the IgM concentration in the NC group, at 1040.95 ± 211.19 pg/mL and 12.85 ± 2.91 pg/mL, respectively (Supplementary Table S2). On the 14th day after infection, the concentrations of LZM and IgM in all infection groups were significantly greater than those in the NC group (*p* ≤ 0.05) (Figure 2). Among the infection groups, the blood serum LZM concentration increased with increasing infection concentrations, reaching a maximum level in the T8 group (1880.95 ± 269.87 pg/mL), but it decreased in the T9 group (1667.84 ± 238.59 pg/mL). The blood serum IgM concentration was highest in the T4 group (18.83 ± 2.59 pg/mL) and decreased with an increasing concentration, indicating that LZM and IgM exhibited different patterns of immune response when common carp were challenged with different concentrations of *Aeromonas veronii*.

### 2.3. Identification and Analysis of DEGs Under Low- and High-Concentration Infection

Since the expression patterns of key genes in head kidney tissues reflect the level of the immune response, we compared differences in the expression profiles of head kidney tissues in response to different concentrations of *Aeromonas veronii* infection. We obtained at least 42,341,064 clean reads from each sample. The average Q20 and Q30 values of the clean reads were 98.67% and 96.44%, respectively, indicating that the sequencing quality of all the samples was satisfactory (Supplementary Table S3).

**Figure 3:**
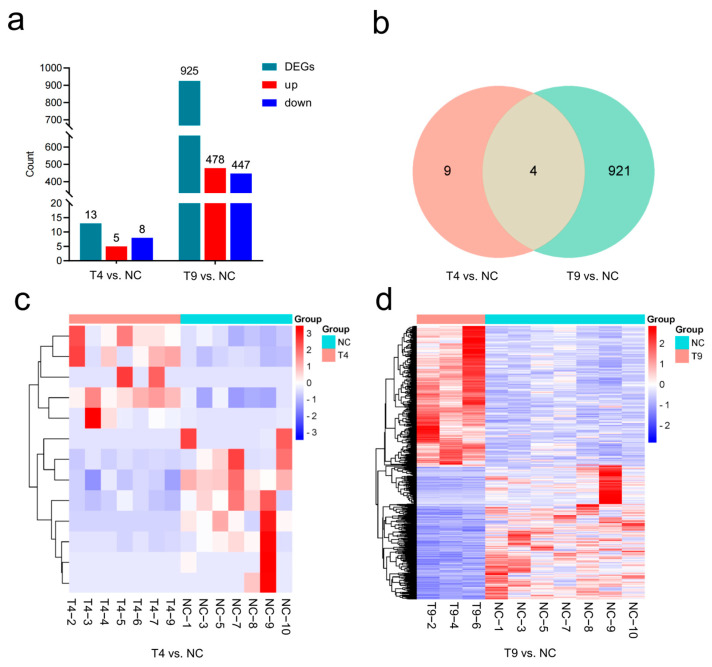
DEGs in the low-concentration and high-concentration groups compared with those in the NC group. (**a**) Statistics of DEGs in two pairs: T4 vs. NC and T9 vs. NC. (**b**) Intersection analysis of differentially expressed genes between T4 vs. NC and T9 vs. NC. (**c**) Heatmap of DEGs in T4 vs. NC: the horizontal coordinate represents the sample number. (**d**) Heatmap of DEGs in the T9 vs. NC: the horizontal coordinate represents the sample number.

Compared with those in the NC group, 13 DEGs were detected in the low-concentration T4 group, including five upregulated and eight downregulated genes (Supplementary Table S4), whereas 925 DEGs were detected in the high-concentration T9 group, including 478 upregulated and 447 downregulated genes (Supplementary Table S5) (Figure 3). The number of DEGs was greater in the high-concentration T9 group than in the low-concentration T4 group, suggesting that a higher concentration of *Aeromonas veronii* elicits a stronger immune response at the gene expression level. In particular, a total of four shared DEGs were detected in two pairs: T4 vs. NC and T9 vs. NC. In addition, nine and 921 unique DEGs remained in the T4 vs. NC and T9 vs. NC pairs, respectively.

### 2.4. Functional and Pathway Enrichment Analyses of the Identified DEGs

**Figure 4:**
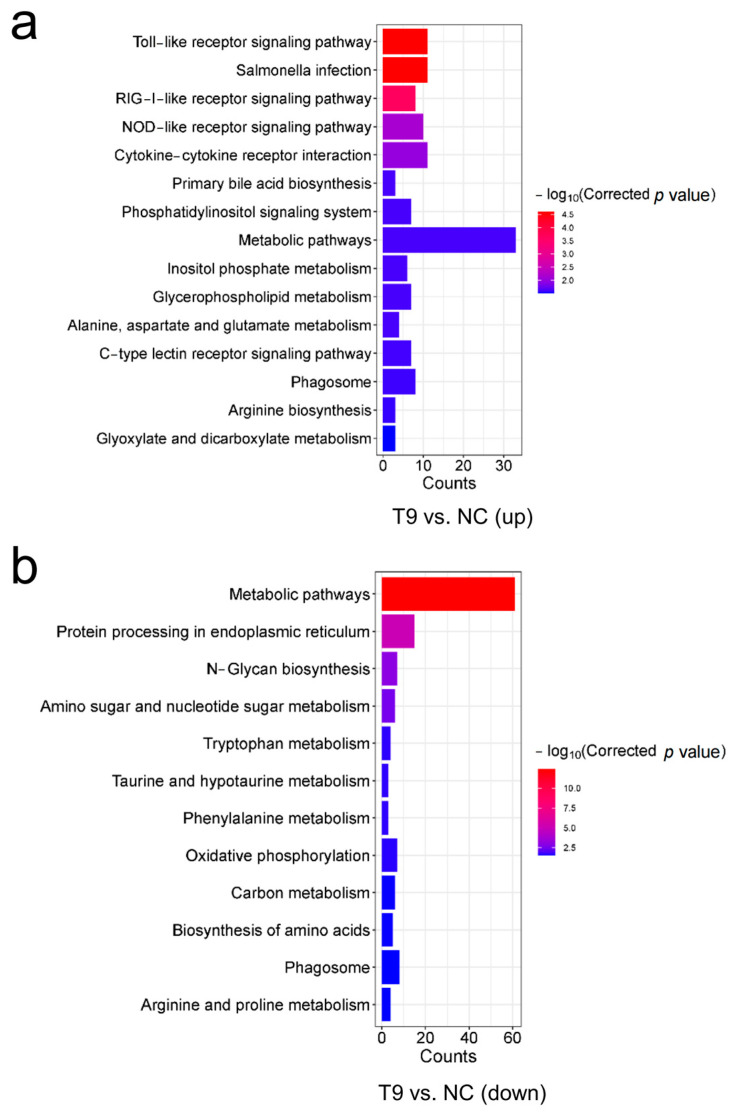
The enriched KEGG terms obtained from KEGG pathway enrichment analysis of the DEGs. (**a**) KEGG terms from enrichment analysis of upregulated genes in T9 vs. NC. (**b**) KEGG terms from enrichment analysis of downregulated genes in T9 vs. NC.

**Figure 5:**
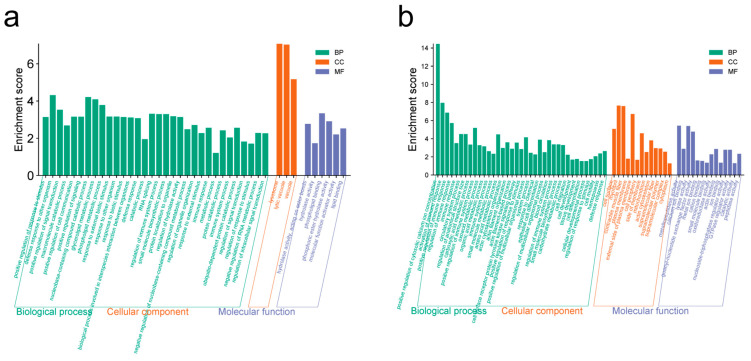
The enriched GO terms obtained from the DEGs. (**a**) GO terms from enrichment analysis of upregulated genes in T9 vs. NC. (**b**) GO terms from enrichment analysis of downregulated genes in T9 vs. NC.

KEGG and GO pathway enrichment analyses were performed to provide a deeper understanding of the functions of the DEGs. A total of 13 DEGs and 925 DEGs obtained from T4 vs. NC and T9 vs. NC groups, respectively, were annotated with KEGG terms. No significant signaling pathways were enriched in the T4 vs. NC group. However, in the T9 vs. NC group, upregulated genes were significantly enriched in 15 KEGG terms, including the Toll−like receptor signaling pathway, the RIG−I−like receptor signaling pathway, the NOD−like receptor signaling pathway, Cytokine−cytokine receptor interaction, the C−type lectin receptor signaling pathway and other important immune signaling pathways (Figure 4b and Supplementary Table S6). Twelve significant KEGG terms were obtained from the downregulated genes, with metabolic pathways being the top significant. (corrected *p* value = 4.10 × 10^−13^) (Figure 4b and Supplementary Table S7). GO enrichment of the DEGs among the upregulated genes in the T9 vs. NC group revealed that 32 out of the top 41 enriched belonged to the biological process category (Figure 5a and Supplementary Table S8). Moreover, among the downregulated genes, 37 of the 64 GO terms belonged to the biological process category (Figure 5b and Supplementary Table S9). The positive regulation of response to stimulus, regulation of the immune system process, immune response, and immune system process were enriched in this category. These pathways play important roles in resistance to *Aeromonas veronii* infection in the head kidney tissues. Apparently, more immune signaling pathways were activated in head kidney tissues of common carp when they were exposed to high concentrations of *Aeromonas veronii*.

### 2.5. WGCNA Revealed the Modules Relevant to Serum LZM and IgM

**Figure 6:**
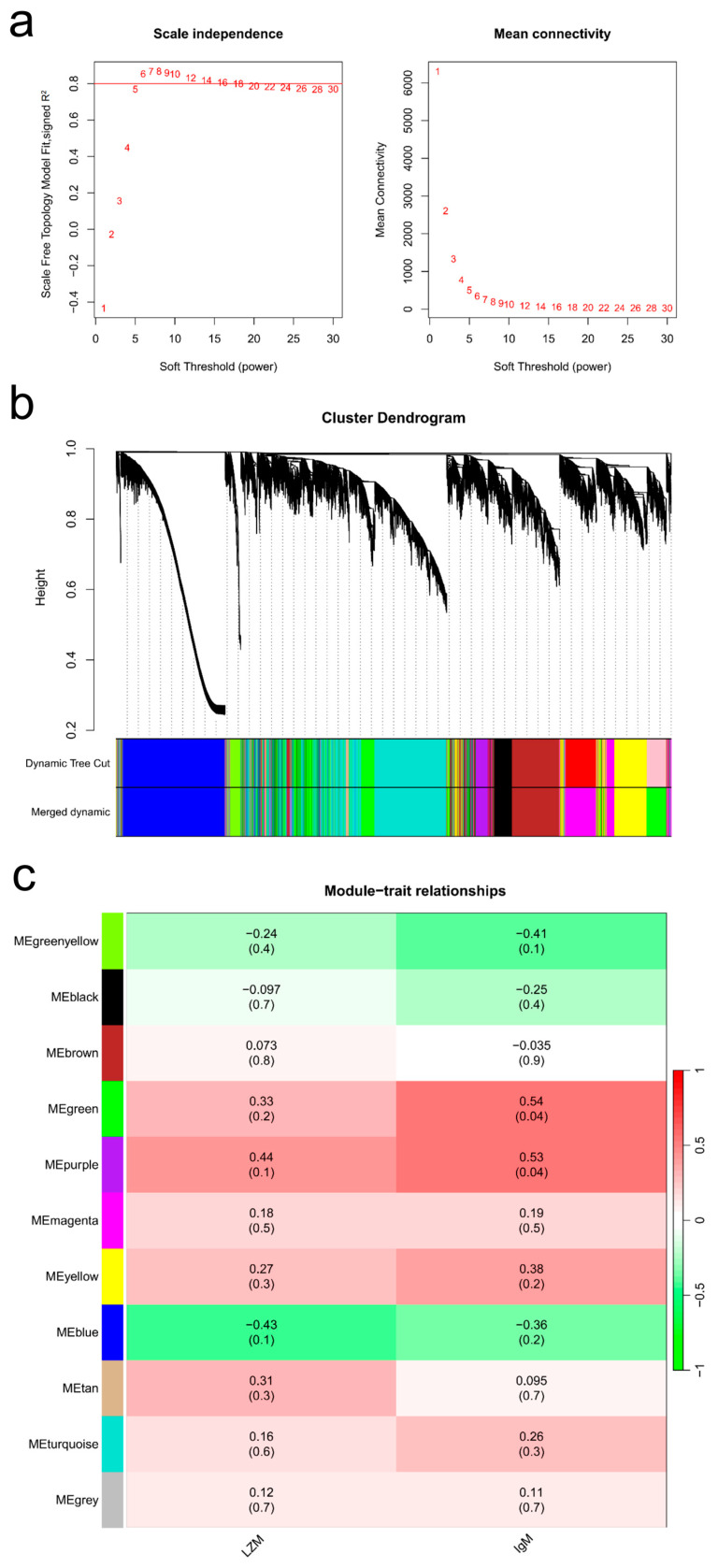
Gene co-expression network analysis of serum LZM and IgM. (**a**) Scale-free topology model and mean connectivity. (**b**) Clustering dendrogram of genes, with dissimilarity based on the topological overlap, together with assigned module colors. The clustered branches represent different modules, and each line represents one gene. (**c**) Module associations with serum LZM and IgM levels, two values in each box; the upper values represent the absolute values of the correlation coefficients, and the lower values represent the *p* values.

WGCNA was performed to investigate the associations between gene modules and particular immune parameters in the serum. After filtering, a total of 15 out of 17 samples and 19,954 out of 43,531 genes were used to construct the co−expression network based on their expressional levels. A scale-free network was subsequently constructed on the basis of the soft threshold, and a clustering dendrogram was generated from gene clustering on topological overlap matrix (TOM) dissimilarity (Figure 6a,b). The correlations between module feature vectors and serum LZM and IgM were analyzed, and no modules were associated with LZM, but two modules were highly correlated with serum IgM: MEgreen (*p* = 0.04, correlation coefficient = 0.54) and MEpurple (*p* = 0.04, correlation coefficient = 0.53) (Figure 6c). Therefore, as modules of interest, the two modules were further studied below.

### 2.6. Functional and Pathway Enrichment Analysis of the Significant Modules and Screening of Hub Genes

**Figure 7:**
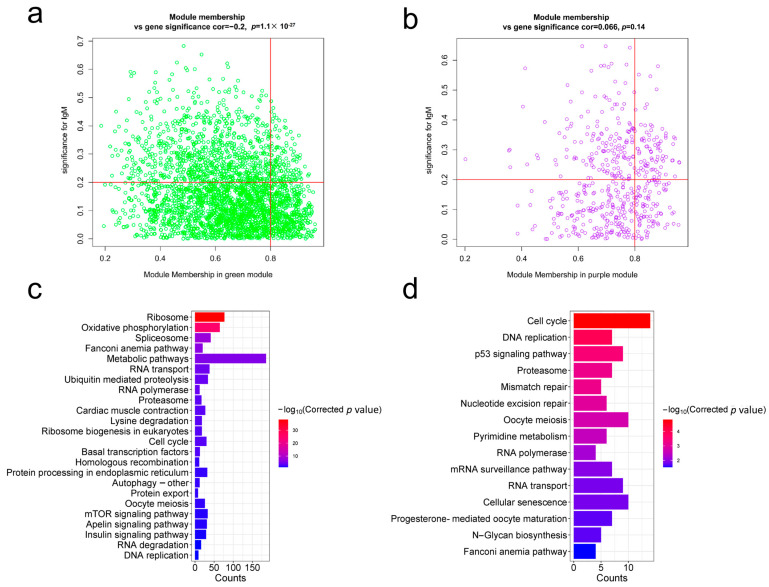
KEGG analysis of genes in the green and purple modules. (**a**) Scatterplot of gene significance and module membership in the green module. The upper right corner represents the hub genes (GS ≥ 0.2 and MM ≥ 0.8). (**b**) Scatterplot of gene significance and module membership in the purple module. The upper right corner represents the hub genes (GS ≥ 0.2 and MM ≥ 0.8). (**c**) Pathway enrichment analysis of all genes in the green module. (**d**) Pathway enrichment analysis of all genes in the purple module.

A total of 2915 and 514 genes were co-expressed in the green and purple modules, respectively (Figure 6a,b). To elucidate the biological significance of these genes, KEGG pathway enrichment revealed that 24 and 15 terms reached a significant level (BH method) (*p* ≤ 0.05) in the two modules. Coincidentally, the cell cycle, DNA replication, proteasome, oocyte meiosis, RNA polymerase, RNA transport and Fanconi anemia pathways were coenriched in two modules (Figure 7c,d) (Supplementary Tables S10 and S11). We subsequently identified 136 hub genes from the 2915 genes in the green module (Supplementary Table S12) and 83 hub genes from the 514 genes in the purple module (Supplementary Table S13), according to gene significance (GS ≥ 0.2) and module membership (MM ≥ 0.8). 

### 2.7. Validation of RNA-Seq Results 

**Figure 8:**
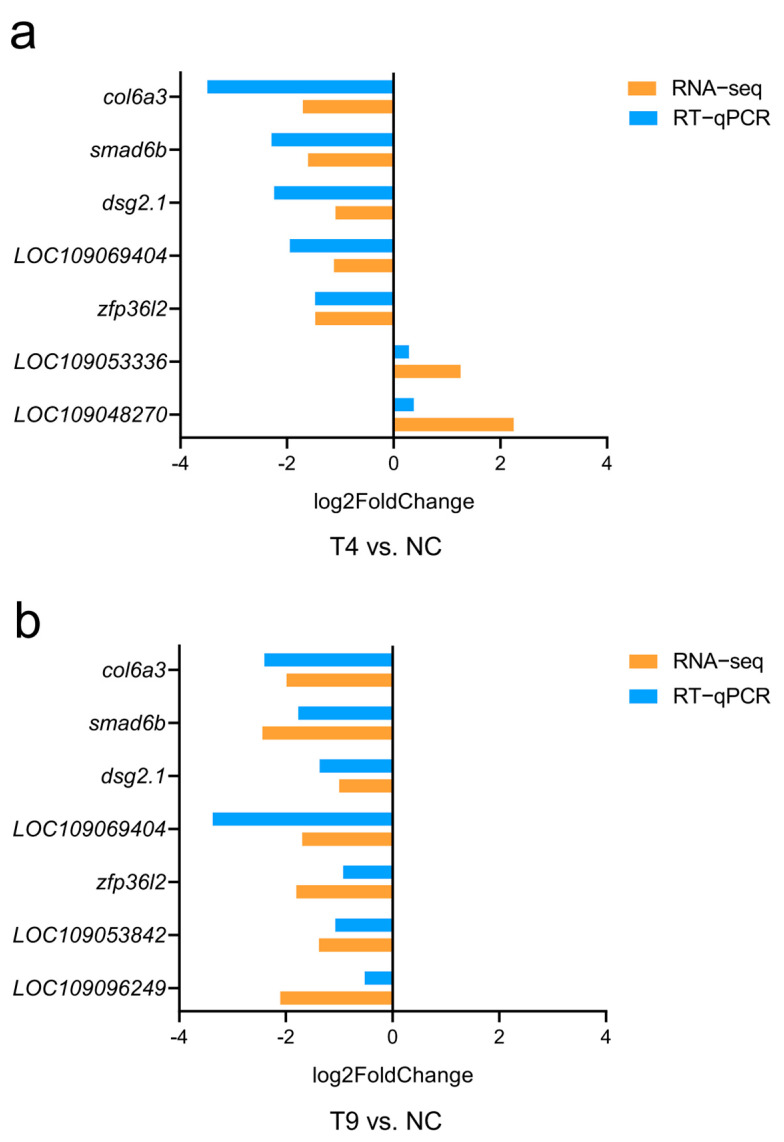
Validation of RNA-Seq results via RT−qPCR. (**a**) Comparison of the results of transcriptome sequencing and RT−qPCR between the T4 and NC groups. (**b**) Comparison of the results of transcriptome sequencing and RT−qPCR between the T9 and NC groups.

To further verify the expression patterns of the genes identified in the RNA−Seq expression analysis and hub genes, we randomly selected five genes for RT−qPCR analysis, namely, *col6a3* (collagen, type VI, alpha 3), *smad6b* (SMAD family member 6b), *dsg2.1* (desmoglein 2, tandem duplicate 1), *LOC109069404* (collagen alpha−2(IV) chain−like) and *zfp36l2* (zinc finger protein 36, C3H type−like 2), and four hub genes, *LOC109053336* (C−C motif chemokine 8−like), *LOC109048270* (SLAM family member 5-like), *LOC109053842* (proteinase−activated receptor 1−like) and *LOC109096249* (collagen alpha−1(IV) chain−like). The same expression trends were found for the *col6a3*, *smad6b*, *dsg2.1*, *LOC109069404* and *zfp36l2* genes in the two pairs. Those genes were downregulated in the treated groups. Only two hub genes, *LOC109053336* and *LOC109048270*, were upregulated. In summary, the RT−qPCR results were consistent with the transcriptome sequencing results, demonstrating the accuracy of the transcriptome sequencing data (Figure 8a,b).

## 3. Discussion

The common carp is a farmed fish species worldwide and an important food source for people, indicating its outstanding economic importance in freshwater aquaculture. Despite their strong environmental adaptability, common carp are still exposed to a variety of pathogens, restricting the development of healthy farming. In particular, the spread of *Aeromonas veronii* is a great threat to the economy in freshwater aquaculture. However, the potential molecular mechanism of immune responses against this pathogen infection remains poorly understood. Some studies have reported the immune response to *Aeromonas veronii* infection in various fish species [22,23,24,25], but these studies are limited in their ability to explain the mechanisms of this pathogen infection in common carp. Our study provides insights into the immune defense mechanisms of common carp in response to infection with different concentrations of *Aeromonas veronii*.

*Aeromonas veronii* can cause high mortality in carp, resulting in obvious symptoms, such as skin ulceration, hemorrhages on the body surface and abdominal and intestinal dropsy in diseased fish, which was similar with previous reports in other freshwater fish [22,24]. In this study, we detected more obvious symptoms and higher mortality under challenge with high concentrations of bacterial suspension. In addition, the diseased fish exhibit a typical inflammatory response, as evidenced by a significant increase in the blood serum LZM and IgM levels. Changes in these two indicators at different infection concentrations have rarely been reported. Notably, LZM, a hydrolytic enzyme present in the body surface mucus, intestinal mucus, serum and macrophages of many fish species, plays an important role in the fish innate immune system and participates in resistance to pathogens such as bacteria, fungi and viruses. IgM is one of the few immunoglobulins found in fish to date and has powerful bactericidal and immunomodulatory effects. Among the infection groups, the trend of increased levels of these two immune factors in the blood serum of diseased fish was different, indicating that there was a time lag between different concentrations of bacteria inducing increases in LZM and IgM. For example, in the T4 group, the level of IgM was higher than that in the other groups at 14 days post-infection, and it is possible that a higher bacterial load triggered the immune response earlier, which coincided with the time of death of the fish in each group.

The head kidney is one of the most important lymphoid tissues in teleosts and plays a crucial role in the immune system against microbial pathogens [26,27]. Changes in gene expression in the head kidney constitute an intrinsic mechanism of response to pathogenic bacterial infection. We compared the DEGs of head kidney tissues subjected to low and high concentrations of *Aeromonas veronii* infection via transcriptome analysis. Compared with those in the control group, greater concentrations of pathogen infection caused more genes to be differentially expressed in head kidney tissues. Pathway enrichment analysis of the DEGs revealed that multiple immune signaling pathways were activated in the host defense system against pathogenic bacterial infection. KEGG and GO analyses revealed the enrichment of the Toll−like receptor signaling pathway, the RIG-I-like receptor signaling pathway, the NOD−like receptor signaling pathway, Cytokine−cytokine receptor interaction, the C−type lectin receptor signaling pathway, the positive regulation of response to stimulus, regulation of immune system process, immune response and immune system process, which was consistent with the results of *Aeromonas veronii* infection in other fish. For example, the Toll-like receptor signaling pathway and Cytokine−cytokine receptor interaction were enriched after *Aeromonas veronii* infection in channel catfish [28]. Transcriptome profiles of the spleen of African catfish (*Clarias gariepinus*) challenged with Aeromonas veronii revealed that NF−kappa B, TNF, NLR, TLR and RLR pathways related to immunity were significantly enriched [18]. In addition, similar immune signaling pathways were activated in *Mastacembelus armatus* during bacterial infection [29].

Changes in the expression of immune-related genes were key factors in regulating these immune signaling pathways. Among the verified genes, *smad6b* is a type of SMAD family member 6 and is involved in the regulation of inflammation. SMAD family member 6, a critical mediator of the TGF-β–BMP pathway, negatively regulates interleukin 1-receptor−Toll−like receptor signals through direct interaction with the adaptor Pellino-1 [30]. *dsg2.1* encodes desmoglein 2, tandem duplicate 1, which is located mainly in the plasma membrane and is involved in homophilic cell adhesion via plasma membrane adhesion molecules [31]. The relationship between desmoglein 2 and inflammation has been reported, for example, intracellular desmoglein 2 cleavage sensitizes epithelial cells to apoptosis in response to proinflammatory cytokines [32]. *zfp36l2* is a zinc finger protein, which is zinc finger protein 36, C3H type−like 2. Zinc finger proteins play important roles in gene regulation. Several zinc finger proteins are crucial for immune regulation because they target mRNAs for degradation and the modulation of signaling pathways [33]. There are no direct reports that the above genes are associated with *Aeromonas veronii* infection in common carp, but our data revealed that the above genes were downregulated in the head kidney after *Aeromonas veronii* infection, suggesting a consistent pattern of the immune response of these genes in the head kidney in response to infection with different concentrations of *Aeromonas veronii*. WGCNA was performed to further identify immune-related genes. Among the hub genes, *LOC109053336*, encoding a C−C motif chemokine 8-like protein, is an inflammatory mediator capable of recruiting immune cells at the site of infection. As a kind of kinase−interacting protein, *akip1* plays a key role in immunological processes. Gao et al. reported that *Akip1* acts as a molecular determinant of cAMP−dependent protein kinase in the NF−kappaB signaling pathway, which plays a crucial role in the pathogenesis of many NF−kappaB−related diseases [34]. *LOC109048270* encodes the signaling lymphocytic activation molecule (SLAM) family member 5−like protein, and SLAM family receptors mediate important regulatory signals in immune cells by binding to members of the SLAM−associated protein (SAP) family of adaptors [35,36].

The host immune response to invading pathogenic bacteria is a systematic and complex biological process. Our study revealed the mechanism underlying the varying degrees of response of common carp to different infectious concentrations of *Aeromonas veronii*, including mortality, blood serum immune factors, and differentially expressed genes in head kidney tissues. Although we found no direct evidence that DEGs or hub genes regulated the immune response in common carp, the biological functions of these genes are closely related to the process of immunity, and further research on these genes is needed.

## 4. Materials and Methods

### 4.1. Sampling and Ethics Statement

This study complied with the regulations of the Animal Care and Use Committee of the Chinese Academy of Fishery Sciences (ACUC-CAFS). A panmictic population was cultured at the Fangshan experimental base of the Chinese Academy of Fishery Sciences (Beijing, China) and fed commercial feed (TongWei, Chengdu, China). A total of 210 half-aged juvenile fish with no skin damage were randomly selected for the experiment (mean body weight: 133.29 ± 26.76 g, mean body length: 17.80 ± 1.21 cm). Prior to the start of the experiment, all the fish were temporarily reared in recirculating water for 7 days at 20 °C.

### 4.2. Bacterial Strains and Infection

*Aeromonas veronii* was used as the infectious strain, which was recorded at China General Microbiological Culture Collection Center (CGMCC 7231). Sterile Luria–Bertani (LB) liquid and LB agarose gel mediums were prepared before bacterial rejuvenation. After the glycerol bacteria preserved at −80 °C were thawed, the tip of a sterilized pipette gun was dipped into the bacterial preservation suspension and then streaked on an LB agar medium and incubated at 28 °C overnight. A single colony was then picked, streaked on new LB plates, and incubated overnight at 28 °C. Then, a single colony was picked and inoculated into an LB liquid medium and incubated overnight at 28 °C with shaking. The subsequently obtained bacterial suspension was measured with an OD = 0.876. Based on the linear correlation between the OD value and concentration (correlation coefficient of 5 × 10^8^), the concentration of the bacterial suspension was calculated to be 4.38 × 10^9^ cfu/mL. The bacterial suspension was diluted 10 times with sterile saline medium. A total of 210 fish were randomly divided into seven groups, with 30 individuals per group. The control group (NC) consisted of 30 fish. The other 180 fish were infected with 4.38 × 10^4^ (T4), 4.38 × 10^5^ (T5), 4.38 × 10^6^ (T6), 4.38 × 10^7^ (T7), 4.38 × 10^8^ (T8), 4.38 × 10^9^ (T9) (cfu/mL) *Aeromonas veronii*, respectively. For each test group, 100 μL of bacterial suspension was injected into the back muscle of each fish. Each sample in the NC group was injected with the same volume of a sterile saline medium. Morbid fish visually exhibited signs of skin ulceration and hemorrhages on the body surface. The experiment lasted for 14 days, and the number of dead individuals in each group during the experiment was recorded. Head kidney tissues were taken from all surviving individuals after the experiment. Before the tissues were collected, all the fish were euthanized with chlorbutanol (0.05%).

### 4.3. Detection of Serum LZM and IgM

On the 14th day post infection, the concentrations of blood serum LZM and IgM in 42 surviving individuals from the NC (*n* = 10), T4 (*n* = 10), T7 (*n* = 6), T8 (*n* = 10) and T9 (*n* = 6) groups were measured via enzyme-linked immunosorbent assay (ELISA) (Bioswamp, Wuhan, China). In brief, standards and samples in duplicate were added to the appropriate Microelisa strip plate wells precoated with an antibody specific to LZM or IgM and combined with the specific antibody. Then, a horseradish peroxidase (HRP)-conjugated antibody specific for LZM or IgM was added to each MicroELISA strip plate well and incubated. After the free components were washed away, TMB substrate suspension was added to each well. Only those wells containing LZM or IgM and HRP-conjugated antibodies appeared blue and then turned yellow after the addition of the stop solution. The optical density (OD) was measured spectrophotometrically at a wavelength of 450 nm. The OD value was proportional to the concentration of LZM or IgM, and the concentrations of LZM or IgM in the samples were calculated by comparing the OD of the samples to the standard curve.

### 4.4. RNA Extraction

To compare the gene expression levels of head kidney tissues in the immune response to *Aeromonas veronii*, we selected the lowest (T4) and highest (T9) concentration groups as representative infected groups for comparative analysis with the NC group to identify differentially expressed genes (DEGs). A total of 17 head kidney tissue samples from the NC (*n* = 7), T4 (*n* = 7) and T9 (*n* = 3) groups were used in the present study. The total RNA of each sample was extracted via a TRIzol^®^ reagent (Invitrogen, Carlsbad, CA, USA). The concentration of RNA was measured with a Nanodrop 2000 (Thermo Fisher Scientific, Wilmington, DE, USA). RNA integrity was assessed with the RNA Nano 6000 Assay Kit of the Agilent 2100 bioanalyzer (Agilent Technologies, CA, USA). The RNA purity was determined via agarose gel electrophoresis.

### 4.5. RNA-Seq Library Construction and Sequencing

For each sample, an mRNA sequencing library with a size of approximately 250–300 bp was generated via an NEBNext^®^ Ultra™ RNA Library Prep Kit for Illumina^®^ (NEB, MA, USA). The library was sequenced on an Illumina platform in 150 bp paired-end mode.

### 4.6. Quality Control and Annotation

We used Trimmomatic to remove the read pairs with adapters, reads containing N and low-quality reads (reads with Qphred ≤ 5 bases accounting for more than 50% of the entire length of the read) [37]. The clean reads were aligned to the reference genome of common carp (GCF_018340385.1) [38] with HISAT2 [39]. StringTie was used to estimate the gene expression levels and generate new transcripts [40]. The new transcripts were annotated by alignment to the Pfam [41] and KEGG [42] databases. The gene expression level was measured as fragments per kilobase of transcript per million fragments mapped (FPKM) [43].

### 4.7. DEGs and Pathway Enrichment Analysis

We identified the differentially expressed genes (DEGs) related to the immune response to *Aeromonas veronii* in T4 and T9 groups, respectively. DEseq2 was used to detect differentially expression genes (DEGs) [44]. The *p* values were corrected via the Benjamini–Hochberg (BH) method [45], and genes with the parameters of corrected *p* value ≤ 0.05 and |log2(fold change)| ≥ 1 were assigned as DEGs. Kyoto Encyclopedia of Genes and Genomes (KEGG) pathway enrichment analysis was applied to identify the functions of the DEGs via KOBAS [46]. We used KOBAS to compare common carp proteins with zebrafish proteins and obtained Gene Ontology (GO) annotations of all the genes. The GO terms of DEGs were obtained with TBtools [47]. A term with a corrected *p* value ≤ 0.05 was considered to be significant.

### 4.8. Gene Co-Expression Network Analysis

The transcriptomes were used to construct a co−expression network via the R package “WGCNA” [48] to identify gene modules closely related to the serum LZM and IgM. The soft−thresholding parameter β was set to 6 on the basis of the criterion of approximate scale−free topology R^2^ ≥ 0.8. The weighted adjacency matrix was transformed into a topological overlap matrix (TOM) to generate networks according to the network connectivity of each gene [49,50,51]. To group genes with similar expression profiles into the same gene modules, we performed average association hierarchical clustering on the basis of TOM-based similarity (1−TOM) with a minimum size cut−off of 100 and a deep split value of 2 for the resulting dendrogram. Dendrites representing highly relevant genes were grouped into a module and labelled with a specific color, and unassigned background genes that did not belong to any module were indicated by gray sections. Module eigengenes (MEs) were referred to as “hub genes” and dependent on degree of module membership (MM) and gene significance (GS), which were the cut-off criteria (|MM| ≥ 0.8 and |GS| ≥ 0.2) [52].

### 4.9. Validating the Gene Expression via Real-Time Quantitative PCR (RT–qPCR)

The genes identified in the RNA−Seq expression analysis and hub genes were randomly selected and validated via RT–qPCR. Total RNA from the head kidney tissues of another nine individuals, including the NC (*n* = 3), T4 (*n* = 3) and T9 (*n* = 3) groups, was extracted with a total RNA rapid extraction kit (BIOFIT, China). cDNA was synthesized with a FastKing one−step degenomic cDNA first strand synthesis premix reagent (TIANGEN, Beijing, China). The specific primers (Table 2) were designed via Primer-BLAST tools at the National Center for Biotechnology Information (NCBI). *β*−*actin* was used as an internal reference gene [53]. RT–qPCR was conducted on a Life Real 9600 thermal cycler using PowerUpTM SYBRTMGreen Master Mix (Applied Biosystems, CA, USA). The thermal cycling conditions were 95 °C for 3 min and 40 cycles of two steps (95 °C for 3 s and 60 °C for 34 s). The relative expression levels of genes between the NC group and the bacterial infection T4 and T9 groups were calculated via the 2^−ΔΔ^CT method [54].

### 4.10. Statistical Analysis

The significant differences between any two groups were determined with one-way analysis of variance (one−way ANOVA). All the results were visualized via R−Project (version 4.2.1) or GraphPad Prism software version 8.0. The data are expressed as the means ± standard deviations (SDs) unless otherwise stated.

## 5. Conclusions

In conclusion, our study revealed that high concentrations of *Aeromonas veronii* infection can cause high mortality and significantly increase the levels of blood serum LZM and IgM in common carp. The transcriptome profiles revealed that the DEGs were involved mainly in immune-related pathways, including the Toll−like receptor signaling pathway, the RIG−I−like receptor signaling pathway, the NOD−like receptor signaling pathway, Cytokine–cytokine receptor interaction, the C−type lectin receptor signaling pathway, the positive regulation of response to stimulus, regulation of immune system process, immune response and immune system processes. Furthermore, we identified 136 and 83 hub genes from the immune-related green and purple modules, respectively. These results revealed the mechanism of the immune response to *Aeromonas veronii* infection in common carp, providing a theoretical foundation for research on disease resistance in fish.

## Figures and Tables

**Table 1:** Morbidity and mortality statistics for the six groups with different pathogen concentrations.

Group	Concentration (cfu/mL)	Total Number	Morbid Individuals	Dead Individuals	Morbidity	Mortality
NC	0	30	0	0	0.00%	0.00%
T4	4.38 × 10^4^	30	0	0	0.00%	0.00%
T5	4.38 × 10^5^	30	2	0	6.67%	0.00%
T6	4.38 × 10^6^	30	2	0	6.67%	0.00%
T7	4.38 × 10^7^	30	22	3	73.33%	10.00%
T8	4.38 × 10^8^	30	29	9	96.67%	30.00%
T9	4.38 × 10^9^	30	29	24	96.67%	80.00%

**Table 2:** Primer sequences for RT–qPCR.

Gene Name	Forward Primer (5′-3′)	Reverse Primer (5′-3′)
*β-actin*	GATGATGAAATTGCCGCACTG	ACCAACCATGACACCCTGATGT
*col6a3*	GCCATCCGTGAGTTTATTCGG	CTTCCACCTTTTGCTGTAAGGC
*smad6b*	GACTTTCCCCCAGCGAAGAA	CGCCACGTTACACCAATGAC
*dsg2.1*	CTTCAAAGACGGCAACAGGC	TCCCCATAGTTCCTTCTGGGA
*zfp36l2*	GTTTGCTCACGGCTATCACG	GAGCCTCGGCTGAGTGTTAC
*LOC109069404*	GGGGACCAGAGGGACCTAAT	CCTGGTGGGCCTTTGAAGAA
*LOC109096249*	TCAAGATGGCTCTCCAGGTTTA	CAAATCTAGCAGGCTCTCCCT
*LOC109053842*	TGTTTCCCATCGCCTCTCTG	TGTCATTTTTGCGTTGCACAT
*LOC109053336*	GAGCTGCTGTAAGGAGGTGT	CGGTCTTTAATCCAGCGCAC
*LOC109048270*	GTGTTGGAGGGAGGTGTTCTC	GTCTAACTGCACTCTGCCTGT

## Data Availability

The transcriptome data reported in this research are available at the Sequence Read Archive (SRA) under accession number PRJNA1128011.

## Supplementary Materials

The following supporting information can be downloaded at: https://www.mdpi.com/article/10.3390/ijms252212070/s1.
